# Resource allocation efficiency in Ethiopian hospitals: technology utilization as mediator and staff competency as moderator

**DOI:** 10.1186/s12962-026-00828-8

**Published:** 2026-09-26

**Authors:** Sintayehu Assefa Yirga

**Affiliations:** College of Business and Economics, Department of Management, Hawassa University, P. O. Box 05, Hawassa City, Ethiopia

**Keywords:** Resource optimization, Patient-centered care, Process efficiency, Resource availability, Technology utilization, Staff competency

## Abstract

**Background:**

Efficient resource allocation remains a major challenge for hospitals in low-resource healthcare systems, where increasing service demand often exceeds the availability of financial, technological, and human resources. Understanding how organizational capabilities improve resource allocation efficiency is therefore essential. This study examined the effects of resource availability, process efficiency, and patient-centered care on resource allocation efficiency in Ethiopian hospitals, while investigating the mediating role of technology utilization and the moderating role of staff competency.

**Methods:**

A quantitative cross-sectional survey was conducted among healthcare professionals working in public and private hospitals in Ethiopia. Data were analyzed using partial least squares structural equation modeling (PLS-SEM) to assess the direct, mediating, and moderating relationships among the study variables. The measurement model was evaluated for reliability and validity, while the structural model was assessed using standardized path coefficients, coefficients of determination (R²), effect sizes (f²), predictive relevance (Q²), and bootstrapping procedures.

**Results:**

The findings indicated that resource availability, process efficiency, and patient-centered care positively and significantly influenced resource allocation efficiency. Technology utilization partially mediated these relationships, demonstrating that greater adoption of digital technologies enhances the efficient use of hospital resources by improving coordination and reducing operational inefficiencies. Staff competency also significantly moderated the relationship between technology utilization and resource allocation efficiency, indicating that the benefits of technology are strengthened when healthcare professionals possess higher levels of knowledge and skills. Overall, the structural model demonstrated substantial explanatory and predictive power for resource allocation efficiency.

**Conclusion:**

The findings suggest that improving resource allocation efficiency requires a balanced investment in both technological capabilities and workforce competency. Hospitals should prioritize digital transformation alongside continuous staff training to maximize the effective utilization of limited healthcare resources. This study extends the healthcare management literature by integrating the Resource-Based View, Socio-Technical Systems Theory, and Dynamic Capabilities Theory to explain how organizational resources, technological capability, and human competency jointly improve resource allocation efficiency in hospitals within a resource-constrained setting.

**Clinical trial number:**

Not applicable.

## Introduction

Ensuring efficient and equitable use of healthcare resources remains a global priority, particularly in low- and middle-income countries where system constraints often compromise patient outcomes. Contemporary healthcare systems increasingly rely on strategic resource availability, streamlined processes, and patient-centered care models to optimize resource allocation [8, 40]. Yet, despite considerable investments, many health organizations continue to struggle with inefficiencies, workforce challenges, and uneven technology adoption. This gap highlights the need to understand not only the direct influence of organizational resources and processes but also the mechanisms and boundary conditions through which these factors translate into better performance.

Emerging evidence suggests that technology utilization plays a pivotal mediating role in bridging resource inputs and operational outcomes [18]. In another interrelated study made by Jeilani and Hussein [44] demonstrate that the adoption of digital health technologies substantially improves healthcare workers’ performance, but only when supportive infrastructure and workload considerations are adequately addressed. Similarly, Wang et al. [61] emphasize that hospital contextual factors such as leadership, staff training, and infrastructure availability critically shape the effectiveness of technology implementation. These findings underscore the notion that technology is not a neutral tool but an enabler whose impact depends on the organizational ecosystem.

Equally important is the role of staff competency as a moderating factor in healthcare transformation. A systematic review by Patil and Shankar [54] confirms that performance expectancy, training, and attitudes strongly determine whether health workers meaningfully adopt digital systems in low-resource settings. Complementing this, Alshammari and Alenezi [9], find that nursing workforce competencies significantly enhance job satisfaction and patient care when integrated with technology. These insights align with socio-technical systems theory, which highlights the interdependence of human and technological subsystems in achieving efficiency and quality.

The dynamic capability perspective further reinforces this relationship, suggesting that organizations must continuously adapt and reconfigure resources to achieve sustainable performance outcomes. Recent studies on AI-driven decision support in hospitals Alves et al. [12] reveal that when staff possess the necessary skills and trust in digital tools, managers are better equipped to optimize resource allocation and reduce inefficiencies. Thus, staff competency not only strengthens the link between processes and outcomes but also determines the effectiveness of technology as a mediator.

Ethiopian healthcare systems continue to struggle with optimizing scarce resources while ensuring high-quality patient care. System-level deficiencies such as weak patient safety culture, limited adoption of evidence-based practices, and inadequate technology use undermine efficiency and increase preventable adverse events [14, 15, 29, 47]. Frontline nurses, particularly in critical care and emergency units, face elevated stress due to resource shortages, high patient demands, and insufficient institutional support [23]. These challenges are further exacerbated by poor integration of patient-centered care and digital technologies, constraining both staff performance and operational efficiency. Although staff competency has the potential to mitigate these pressures, its role in enhancing resource allocation and patient outcomes remains underexplored. This study, therefore, investigates how staff competency, technology utilization, and patient-centered care collectively influence process efficiency and resource optimization, providing critical insights to strengthen Ethiopia’s healthcare delivery system.

To sum it up, these studies highlight the importance of integrating resource-based, socio-technical, and dynamic capability perspectives to understand healthcare performance. Building on this foundation, the present study investigates how resource availability, process efficiency, and patient-centered care influence the optimization of resource allocation, while considering the mediating role of technology utilization and the moderating effect of staff competency. By examining these relationships in a resource-constrained healthcare context, the study contributes to both theoretical advancement and practical strategies for sustainable healthcare delivery.

## Theoretical underpinning

This study is anchored in three complementary theoretical perspectives Resource-Based View (RBV), Socio-Technical Systems (STS) theory, and Dynamic Capabilities theory which together provide a robust lens for understanding how hospitals optimize resource allocation.

Resource-Based View (RBV). RBV suggests that organizations achieve superior performance by leveraging resources that are valuable, rare, inimitable, and non-substitutable. In healthcare, staff competency represents a vital human resource, while technology utilization reflects a strategic capability that can enhance efficiency. When hospitals combine skilled personnel with effective technology, they are better positioned to reduce inefficiencies and allocate scarce resources more effectively. Empirical evidence supports this view, showing that healthcare institutions with well-trained staff and strong technological capacity are more likely to improve operational efficiency and patient outcomes [34].

Socio-Technical Systems (STS) theory. STS emphasizes the interdependence between people and technology in shaping organizational outcomes. In this study, technology utilization is conceptualized as a mediator, reflecting the mechanism by which inputs such as process efficiency, resource availability, and patient-centered care are transformed into optimized resource allocation. Hospitals adopting digital systems without adequate staff training or alignment with workflows often fail to realize expected benefits, underscoring the need for socio-technical integration. Prior studies confirm that the effectiveness of health IT depends not only on technological investment but also on leadership, training, and workflow alignment [62].

Dynamic Capabilities theory. Building on RBV, the dynamic capabilities perspective highlights the ability of organizations to adapt, reconfigure, and renew resources in response to changing environments (Teece et al., 1997). In resource-constrained settings such as Ethiopian hospitals, staff competency acts as a moderator, shaping the strength of the relationship between organizational inputs and resource allocation outcomes. Competent staff can adapt processes, maximize the use of technology, and ensure that scarce resources are redirected to areas of greatest need [13]. Studies mas de by Al-Amin et al. [5] applying dynamic capabilities in healthcare show that adaptability, learning, and resource reconfiguration are critical for sustaining efficiency and service quality.

**Fig. 1:**
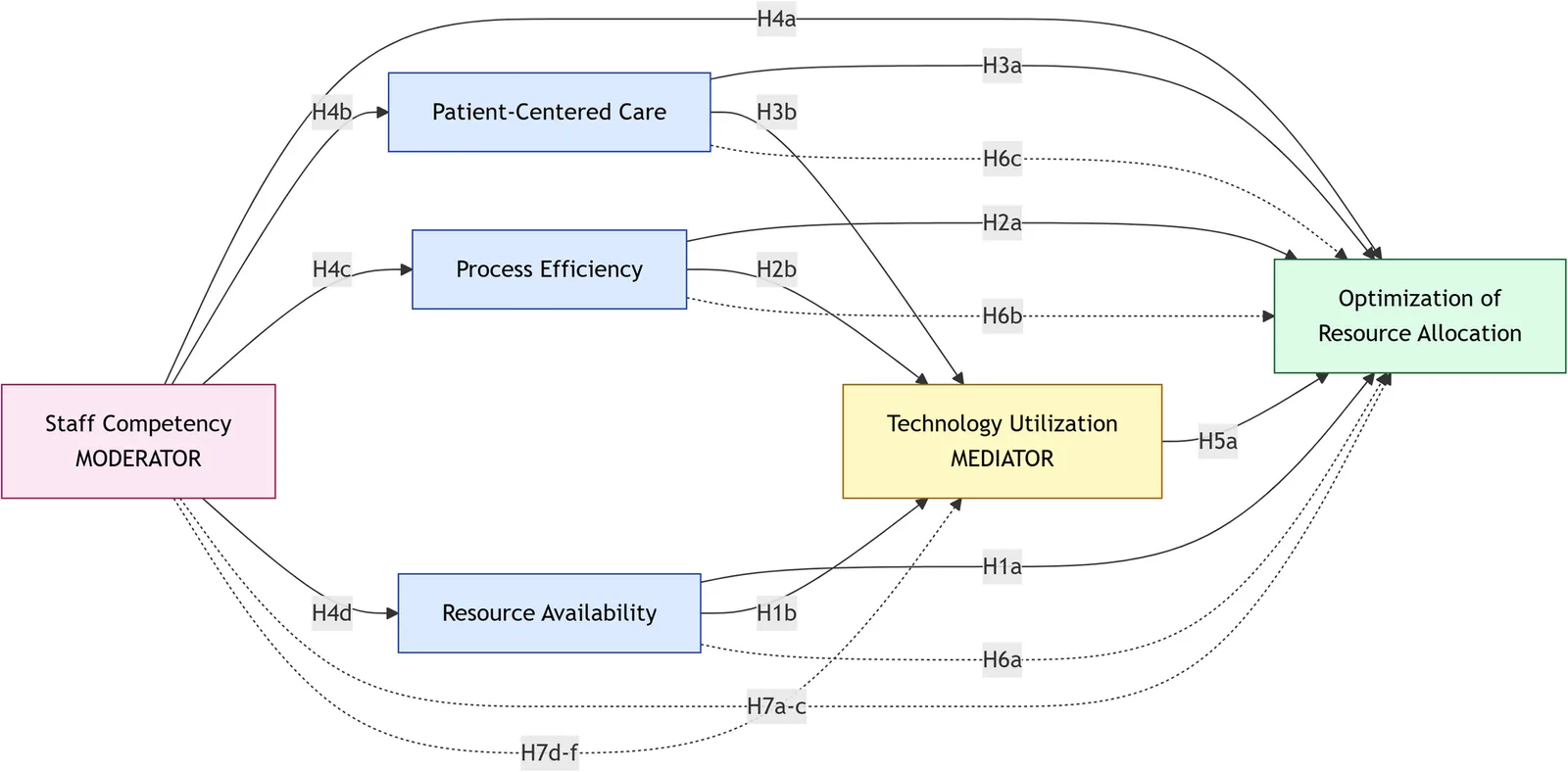
Research model

Together these theories justify the model tested in this paper (shown in Fig. 1): (a) RBV explains why staff competency and technology are strategic assets that matter for resource optimization; (b) STS explains the mediating role of technology utilization how inputs and process changes become effective through socio-technical integration; and (c) Dynamic Capabilities explain why competency and technology must be continuously redeployed to sustain optimized allocation under changing conditions. Empirical work on EHRs and digital tools shows measurable gains in efficiency and allocation when implementation is well managed, reinforcing the theoretical claims [24].

### Hypotheses development

#### Resource availability and optimization of resource allocation

At the core of any healthcare system is the accessibility of essential resources whether equipment, medications, or workforce. Without sufficient resources, even the most efficient processes or skilled staff cannot function optimally. Resource availability therefore acts as a foundation for effective service delivery [38]. When resources are adequately supplied and equitably distributed, hospitals can minimize delays, enhance treatment accuracy, and improve patient flow. Study by Gabriel [37] demonstrated that availability of resources significantly influences not only operational efficiency but also patient outcomes. Based on this review, the following hypothesis were framed:

##### H1a

Resource availability is positively associated with optimization of resource allocation.

##### H1b

Resource availability is positively associated with technology utilization.

#### Process efficiency and optimization of resource allocation

Efficient processes ensure that healthcare services are delivered without unnecessary duplication, bottlenecks, or waste. Process efficiency involves streamlining workflows, reducing waiting times, and standardizing procedures so that patients receive timely care. Nugroho et al. [49] disclosed that when processes are optimized, resources such as staff hours, medical equipment, and facilities are used more judiciously, contributing to overall system sustainability. Empirical evidence highlights that hospitals adopting lean process strategies not only reduce costs but also improve service quality, thereby achieving better resource utilization [39]. Based on this review, the following hypothesis were framed:

##### H2a

Process efficiency is positively associated with optimization of resource allocation.

##### H2b

Process efficiency is positively associated with technology utilization.

#### Patient-centered care and optimization of resource allocation

Patient-centered care emphasizes responsiveness to patients’ needs, values, and preferences. While this might appear primarily as a humanistic goal, it also has a direct effect on resource utilization [19, 28, 65]. In another study made by Fionda et al. [35] when care plans are tailored to patients, unnecessary tests, repeated treatments, and avoidable admissions are minimized, leading to more rational use of limited resources. Moreover, Al-Assaf et al. [6] disclosed that engaged patients are more likely to adhere to treatment, reducing complications and the need for additional interventions. Recent empirical evidence shows that prioritizing patient-centered approaches enhances both satisfaction and the efficiency of healthcare delivery [53, 58]. Based on this review, the following hypothesis were framed:

##### H3a

Patient-centered care is positively associated with optimization of resource allocation.

##### H3b

Patient-centered care is positively associated with technology utilization.

### Staff competency and healthcare outcomes

Healthcare systems depend heavily on the skills and expertise of their staff. Competent professionals not only carry out their tasks effectively but also anticipate problems, adapt to challenges, and use resources wisely [52]. When staff demonstrate strong clinical and managerial skills, hospitals are more likely to optimize the allocation of resources ensuring that equipment, medications, and workforce capacity are used where they are most needed. Studies confirm that well-trained staff reduce inefficiencies and contribute directly to better organizational outcomes. Beyond direct efficiency, staff competency also shapes the patient experience [59]. Compassionate, knowledgeable professionals engage patients more effectively, communicate clearly, and provide care tailored to individual needs. This strengthens patient-centered care, making patients feel heard and valued. In addition, skilled staff tend to streamline workflows and minimize unnecessary steps, thereby enhancing process efficiency [2]. Their ability to coordinate across departments and adopt best practices ensures that care delivery is both timely and effective. Finally, competent staff are better positioned to identify resource gaps, monitor utilization, and advocate for appropriate supplies. Their judgment and experience contribute to stronger resource availability, since they help prevent stock outs, misuse, or underutilization of key assets. Based on this review, the following hypothesis were formulated:

#### H4a

Staff competency is positively associated with optimization of resource allocation.

#### H4b

Staff competency is positively associated with patient-centered care.

#### H4c

Staff competency is positively associated with process efficiency.

#### H4d

Staff competency is positively associated with resource availability.

### Technology utilization and optimization of resource allocation

In today’s healthcare environment, where resources are often stretched thin, technology has become more than just a supporting tool it is a driver of smarter decision-making and efficient care delivery. The use of digital platforms such as electronic health records, telemedicine, and predictive analytics allows hospitals to see in real time where resources are needed most and to allocate them with greater precision [51]. Research consistently shows that when healthcare providers integrate technology into their daily operations, they achieve better workflow efficiency, fewer redundancies, and more transparent tracking of resources and this not only improves the use of existing capacity but also builds trust, since patients and provider’s alike benefit from fairer and more accountable distribution of limited resources [48, 64]. From a broader systems perspective, technology empowers staff to make faster, evidence-based decisions, which strengthens the entire resource allocation process. Based on this review, the following hypothesis were framed:

#### H5a

 Technology utilization is positively associated with optimization of resource allocation.

#### Technology utilization as a mediator

Digital tools such as electronic health records, decision-support systems, and predictive analytics serve as enabling mechanisms that translate resources, efficient processes, and patient-centered practices into tangible improvements in allocation [32, 43]. Recent study made by Binsar et al., [22] indicates that technology enhances coordination, prevents duplication, and supports data-driven resource management. Therefore, technology utilization is hypothesized to mediate the relationships between the independent variables and optimization of resource allocation. Based on this review, the following hypothesis were framed:

##### H6a

Technology utilization mediates the relationships between resource availability and optimization of resource allocation.

##### H6b

Technology utilization mediates the relationships between process efficiency and optimization of resource allocation.

##### H6c

Technology utilization mediates the relationships between patient-centered care and optimization of resource allocation.

#### Staff competency as a moderator

While technology offers potential benefits, its effectiveness depends heavily on the skills and competencies of healthcare staff. Skilled professionals are better able to integrate digital tools into workflows, interpret data, and use information systems to support decision-making [41]. Evidence shows that staff competency strengthens the positive impact of technology on performance outcomes, while low competency can diminish these benefits [20]. Thus, staff competency is expected to moderate the relationship between technology utilization and optimization of resource allocation. Based on this review, the following hypothesis were framed:

##### H7a

Staff competency positively moderates the relationship between resource availability and optimization of resource allocation.

##### H7b

Staff competency positively moderates the relationship between process efficiency and optimization of resource allocation.

##### H7c

Staff competency positively moderates the relationship between patient-centered care and optimization of resource allocation.

##### H7d

Staff competency positively moderates the relationship between resource availability and technology utilization.

##### H7e

Staff competency positively moderates the relationship between process efficiency and technology utilization.

##### H7f

Staff competency positively moderates the relationship between patient-centered care and technology utilization.

### Research design

This study employed a quantitative, cross-sectional survey design, which is widely recognized for examining relationships among organizational and healthcare-related variables within real-world settings. Such an approach allows for the systematic testing of hypothesized relationships between independent variables (resource availability, process efficiency, and patient-centered care) and the dependent variable (optimization of resource allocation), while also considering the mediating role of technology utilization and the moderating effect of staff competency. The cross-sectional design has been shown to be particularly effective in healthcare management studies, offering a snapshot of current practices and enabling comparisons across institutions [10].

### Population and sampling

The target population for this study comprised healthcare professionals working in both government and private hospitals in Sidama Regional State, Ethiopia. This group was considered appropriate because they are directly involved in day-to-day patient care, administrative decision-making, and the use of hospital resources, making them best positioned to provide relevant insights into how technology and staff competency shape the optimization of healthcare resource allocation.

### Sample size determination

The sample size was determined using Cochran’s formula [27], which is widely applied in social science and healthcare studies:

$${n}_{0}=\frac{{Z}^{2}\cdot{p}\cdot(1-{p})}{{e}^{2}}$$

**Where**:

- n_0_ = sample size.
- Z = Z-value (1.96 for 95% confidence level).
- p = estimated proportion of the population (0.5, maximizing variability).
- e = margin of error (0.05).

Substituting values:

$${n}_{0}=\frac{{(1.96)}^{2}\cdot{0.5}\cdot(1-{0.5})}{{(0.05)}^{2}}=384$$

Given resource constraints and to ensure feasibility of data collection, the sample size was adjusted to 350 respondents, which remains statistically robust for multivariate analysis.

### Sampling technique and distribution

A stratified random sampling approach was employed (see Table 1). Hospitals were first stratified by ownership type (government vs. private), and proportional allocation was applied to reflect the real distribution of institutions in Sidama. Within each stratum, staff were further classified into four professional categories (nurses, physicians, administrative staff, and ICT personnel), and proportional random sampling was used.

**Table 1:** Sample distribution across hospital ownership and staff categories

Hospital Ownership	Nurses (55%)	Physicians (20%)	Admin Staff (15%)	ICT Staff (10%)	Total
Government (65%)	125	45	34	23	227
Private (35%)	67	23	17	16	123
**Total**	**192**	**68**	**51**	**39**	**350**

This multi-stage stratified random sampling ensured fair representation of both ownership types and professional groups, thereby enhancing the external validity and generalizability of the findings. In deciding on a sample size and sampling strategy, similar studies in Ethiopia have provided useful precedents. For example, these studies [14, 15] used stratified simple random sampling across hospitals and staff categories to ensure representative coverage of healthcare providers and achieved strong analytical power. Mekonnen et al. [47] conducted a cross-sectional survey with approximately 480 staff members in Ethiopian public hospitals, yielding reliable scales (Cronbach’s α ~ 0.77). Likewise, Dessie et al. [29] stratified their sample by staff type (nurses, physicians, administrative, etc.) to achieve meaningful comparisons across roles. These examples support the methodological decisions in this study using Cochran’s sample size formula to determine an adequate base sample, adjusting downward to 350 for feasibility, and applying stratified random sampling to ensure representation across hospital types and staff categories.

### Common method bias assessment

Because all constructs were measured using a single questionnaire administered to the same respondents, the potential influence of common method bias (CMB) was assessed. Procedurally, several measures were implemented to minimize CMB, including ensuring respondent anonymity and confidentiality, emphasizing voluntary participation, using clear and contextually appropriate measurement items, and carefully organizing questionnaire items to reduce response consistency bias.

Statistically, Harman’s single-factor test was conducted by loading all measurement items into an exploratory factor analysis. The results showed that the first factor explained 22.1% of the total variance, which is below the recommended 50% threshold, indicating that common method bias was not a significant concern. Additionally, full collinearity testing using variance inflation factor (VIF) values was conducted, and all constructs recorded values below the conservative threshold of 3.3. These results provide evidence that common method bias is unlikely to have substantially affected the relationships among the study variables. Therefore, the findings of the study can be considered robust against potential common method bias.

### Measurement

Measurement of constructs was based on validated scales adapted from prior peer-reviewed studies [17, 46, 55]. Resource availability, process efficiency, patient-centered care, staff competency, and technology utilization were measured using multi-item Likert scales, each reflecting widely accepted dimensions from healthcare operations and management literature. This ensured both reliability and validity of the measures. Before full deployment, the questionnaire underwent a pilot test to refine clarity, appropriateness, and contextual fit, following recommendations in prior empirical research [25, 26].

### Method of analysis

The collected data were analyzed using a two-stage analytical approach based on Partial Least Squares Structural Equation Modeling (PLS-SEM), which is appropriate for examining complex models involving multiple latent constructs, mediation effects, and moderation relationships. The analysis was conducted using SmartPLS 4.0. The choice of PLS-SEM was justified by the exploratory and predictive nature of the study, the presence of multiple theoretical relationships, and the objective of assessing both direct and indirect effects among the study constructs.

In the first stage, the measurement model was assessed to evaluate the reliability and validity of the constructs. Indicator reliability was examined by assessing outer loadings, with values above the recommended threshold of 0.70 considered acceptable. Internal consistency reliability was evaluated using Cronbach’s alpha, rho_A, and composite reliability (CR), where values exceeding 0.70 indicated satisfactory reliability. Convergent validity was assessed through the average variance extracted (AVE), with values above 0.50 confirming that the constructs explained more than half of the variance of their respective indicators. Discriminant validity was examined using the heterotrait–monotrait ratio (HTMT) criterion and the Fornell–Larcker criterion to ensure that the constructs were empirically distinct from one another.

In the second stage, the structural model was evaluated by examining the hypothesized relationships among the constructs. The significance of the structural paths was tested using a bootstrapping procedure with 5,000 resamples to generate standard errors, t-values, p-values, and confidence intervals. The explanatory power of the model was assessed using the coefficient of determination (R²), while the predictive relevance of the model was evaluated using Stone–Geisser’s Q² values obtained through the blindfolding procedure. Effect sizes (f²) were also examined to determine the practical contribution of each exogenous construct to the endogenous variables.

To assess the mediation effect of technology utilization, the indirect effects were tested using bootstrapping procedures. Mediation was established when the indirect effect was statistically significant based on the confidence intervals, following contemporary recommendations for mediation analysis in PLS-SEM. The direct effect of the predictors on resource allocation efficiency was also examined to determine whether the mediation effect was partial or full.

Furthermore, the moderating effect of staff competency was assessed by creating an interaction term between the relevant predictor variable(s) and staff competency. The significance of the interaction effect was evaluated through bootstrapping, and the strength and direction of the moderation relationship were interpreted based on the significance of the interaction coefficient and the change in explained variance.

Finally, additional diagnostic analyses were conducted to ensure the robustness of the findings. Multicollinearity among predictor constructs was assessed using the variance inflation factor (VIF), with values below the recommended threshold indicating that multicollinearity was not a concern. Common method bias was also assessed using procedural remedies and Harman’s single-factor test. The results confirmed that common method bias was unlikely to significantly influence the observed relationships.

## Results

This section presents the empirical findings of the study, focusing on the relationships among resource availability, process efficiency, and patient-centered care in driving the optimization of resource allocation. The analysis further explores the mediating role of technology utilization and the moderating effect of staff competency in strengthening or weakening these relationships. Both measurement and structural models were examined to ensure reliability, validity, and the robustness of the hypothesized paths. The results not only highlight significant associations between the key constructs but also shed light on the mechanisms through which healthcare organizations can enhance resource allocation efficiency in resource-constrained environments.

**Table 2:** Summary results of the measurement model

Constructs	Items	Loadings	Cronbach’s Alpha (CA)	Rho_A	Composite Reliability (CR)	AVE	Variance Inflation Factor (VIF)
Optimization of Resources	OR1	**0.734**					**1.566**
	OR2	**0.785**					**1.686**
	OR3	**0.748**	**0.810**	**0.814**	**0.868**	**0.56**	**1.654**
	OR4	**0.731**				**7**	**1.613**
	OR5	**0.767**					**1.762**
Patient Centered Care	PC1	**0.923**					**3.039**
	PC2	**0.876**					**2.476**
	PC3	**0.903**	**0.904**	**0.908**	**0.933**	**0.77**	**3.042**
	PC4	**0.820**				**7**	**1.966**
Process Efficiency	PE1	**0.765**					**1.425**
	PE2	**0.719**					**1.451**
	PE3	**0.706**	**0.729**	**0.743**	**0.829**	**0.54**	**1.384**
	PE4	**0.768**				**7**	**1.306**
Resource Availability	RA1	**0.815**					**1.794**
	RA2	**0.904**					**2.941**
	RA3	**0.873**	**0.846**	**0.861**	**0.897**	**0.68**	**2.674**
	RA4	**0.713**				**8**	**1.451**
Staff Competency	SC1	**0.740**					**1.427**
	SC2	**0.753**					**1.423**
	SC3	**0.821**	**0.777**	**0.779**	**0.857**	**0.60**	**1.784**
	SC4	**0.783**				**0**	**1.598**
Technology Utilization	TU1	**0.764**					**1.685**
	TU2	**0.853**					**2.126**
	TU3	**0.795**				**0.673**	**1.775**
	TU4	**0.864**	**0.837**	**0.840**	**0.891**		**2.234**

**Fig. 2:**
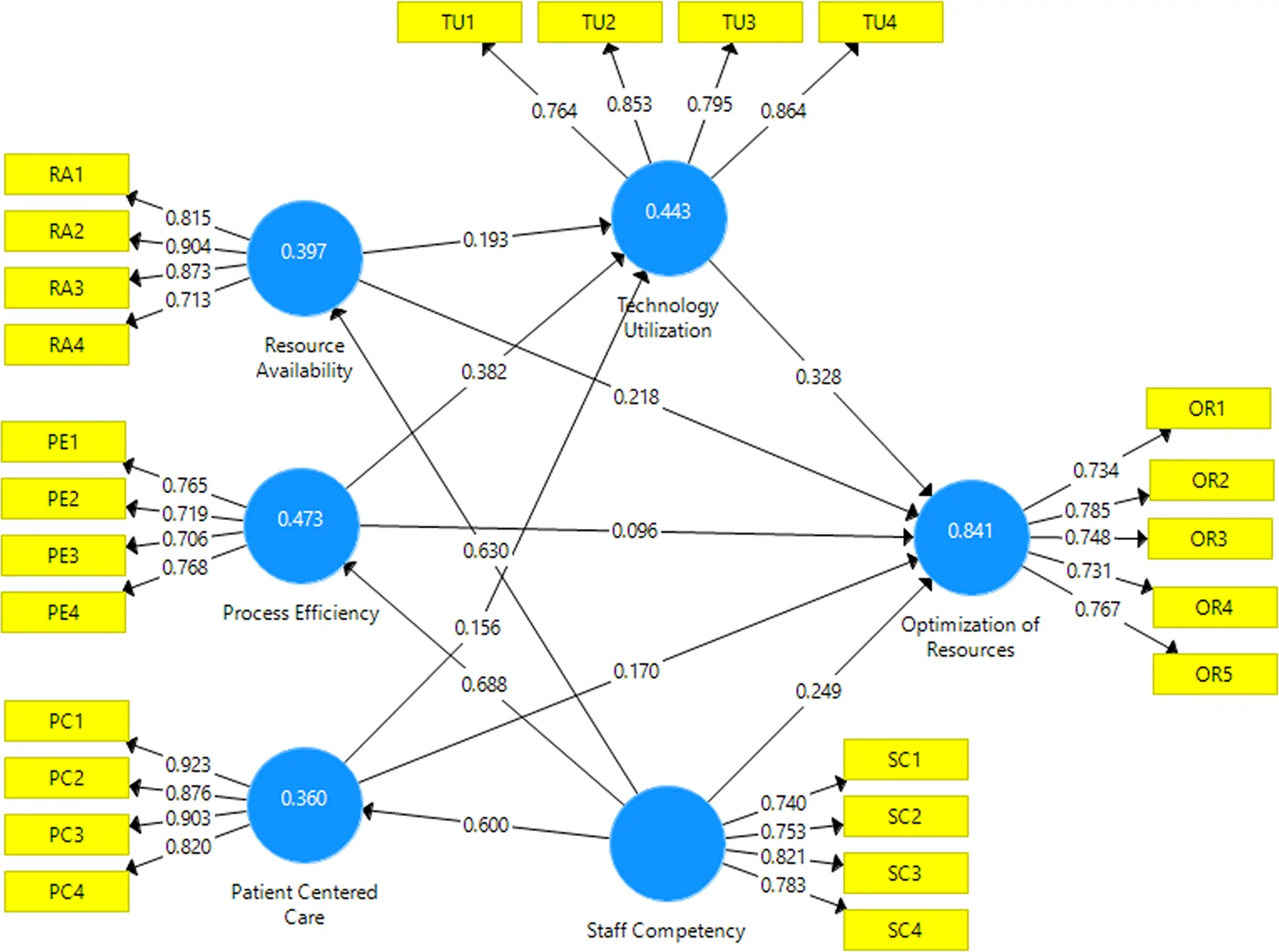
Measurement model

Table 2 and Fig. 2 present the reliability and validity assessment of the study constructs. The results show that all factor loadings exceed the recommended minimum value of 0.70, confirming that the items are good indicators of their respective constructs. Cronbach’s Alpha (CA), rho_A, and Composite Reliability (CR) values for all variables are above the 0.70 threshold, indicating strong internal consistency and scale reliability. In addition, the Average Variance Extracted (AVE) values range from 0.547 to 0.777, all surpassing the 0.50 benchmark, thereby establishing convergent validity. Among the constructs, patient-centered care demonstrates the highest reliability (CR = 0.933, AVE = 0.777), while process efficiency shows the lowest AVE (0.547), though still within acceptable limits. Overall, the results confirm that the measurement model in Table 2 and Fig. 2 meets the required psychometric standards, ensuring that the constructs are appropriate for subsequent structural model analysis.

The Variance Inflation Factor (VIF) values for all measurement items range from 1.306 to 3.042, which are well below the recommended threshold of 5.0 for assessing multicollinearity in PLS-SEM. Moreover, all VIF values are below the more stringent cutoff of 3.3 suggested by Kock [45] for evaluating full collinearity and potential common method bias. These results indicate that multicollinearity among the indicators is not a concern, and no evidence of common method bias is detected. Consequently, the measurement model demonstrates adequate indicator independence, supporting the robustness and stability of the estimated parameters. Overall, these results confirm that the measurement model is reliable and valid, providing a robust foundation for testing the proposed moderation effects in the structural model.

**Table 3:** Discriminant validity of Fornell-Larcker criterion

Constructs	OR	PC	PE	RA	SC	TU
Optimization of Resources	0.753					
Patient Centered Care	0.630	0.882				
Process Efficiency	0.550	0.464	0.740			
Resource Availability	0.471	0.554	0.548	0.829		
Staff Competency	0.332	0.400	0.488	0.430	0.775	
Technology Utilization	0.223	0.355	0.330	0.397	0.546	0.820

**Table 4:** Discriminant validity of Heterotrait-Monotrait ratio (HTMT) criteria

Constructs	OR	PC	PE	RA	SC	TU
Optimization of Resources						
Patient Centered Care	**0.635**					
Process Efficiency	**0.343**	**0.685**				
Resource Availability	**0.415**	0.454	**0.643**			
Staff Competency	**0.339**	**0.316**	0.585	**0.772**		
Technology Utilization	**0.532**	**0.235**	**0.483**	**0.505**	**0.645**	

Tables 3 and 4 demonstrate that discriminant validity was satisfactorily established using both the Fornell–Larcker criterion and the Heterotrait–Monotrait Ratio (HTMT). According to the Fornell–Larcker criterion (Table 3), the square root of the Average Variance Extracted (AVE) for each construct (diagonal elements) is greater than its correlations with all other constructs (off-diagonal elements). For example, the diagonal values for Optimization of Resources (0.753), Patient-Centered Care (0.882), Process Efficiency (0.740), Resource Availability (0.829), Staff Competency (0.775), and Technology Utilization (0.820) all exceed their corresponding inter-construct correlations, indicating that each construct shares more variance with its own indicators than with other latent constructs [36]. Furthermore, the HTMT results presented in Table 4 show that all pairwise HTMT values range from 0.235 to 0.772, which are well below the recommended threshold of 0.85 [42]. The highest HTMT value (0.772 between Resource Availability and Staff Competency) remains within the acceptable limit, providing additional evidence that the constructs are empirically distinct. Collectively, these findings confirm that the measurement model satisfies the requirements for discriminant validity and that each construct captures a unique theoretical concept without evidence of construct overlap or redundancy.

**Table 5:** Coefficient of determination (R^2^), and predictive relevance (Q^2^) and effect size (F^2^)

Exogenous Constructs	F^2^			*R* ^2^	*R*^2^ Adjusted	Q^2^
	OR	TU	SC			
**Optimization of Resources**				**0.841**	**0.839**	**0.354**
**Technology Utilization**	**0.187**			**0.443**	**0.438**	**0.451**
**Staff Competency**	**0.094**			**0.640**	**0.637**	**0.325**
**Process Efficiency**	**0.021**	**0.110**	**0.899**	**0.473**	**0.472**	**0.239**
**Resource Availability**	**0.094**	**0.022**	**0.659**	**0.397**	**0.395**	**0.482**
**Patient Centered Care**	**0.072**	**0.020**	**0.562**	**0.360**	**0.358**	**0.581**

### Coefficient of determination (R²), predictive relevance (Q²), and effect size (f²)

The explanatory power and predictive relevance of the structural model were assessed using the coefficient of determination (R²), adjusted R², predictive relevance (Q²), and effect size (f²), as presented in Table 5. The results indicate that the model demonstrates substantial explanatory power for the endogenous constructs. Specifically, Optimization of Resources showed a high level of explained variance (R² = 0.841; adjusted R² = 0.839), indicating that the predictor variables explain 84.1% of the variance in optimization of resources. Similarly, Technology Utilization (R² = 0.443; adjusted R² = 0.438) and Staff Competency (R² = 0.640; adjusted R² = 0.637) demonstrated moderate to substantial explanatory power.

The predictive relevance of the model was evaluated using Stone–Geisser’s Q² values. All endogenous constructs reported Q² values greater than zero, confirming the model’s predictive relevance. Specifically, Optimization of Resources (Q² = 0.354), Technology Utilization (Q² = 0.451), Staff Competency (Q² = 0.325), Process Efficiency (Q² = 0.239), Resource Availability (Q² = 0.482), and Patient-Centered Care (Q² = 0.581) demonstrated satisfactory predictive capability.

The effect sizes (f²) were examined to determine the relative contribution of each exogenous construct to the endogenous variables. The results indicate that the magnitude of predictor effects varied across relationships. For example, Process Efficiency demonstrated a strong effect on Staff Competency (f² = 0.899), whereas other relationships showed small to moderate effect sizes. These findings suggest that although all proposed relationships contribute to explaining the endogenous constructs, their practical influence differs in magnitude. Overall, the R², Q², and f² results confirm that the proposed structural model possesses adequate explanatory power, predictive relevance, and meaningful effect sizes, supporting the robustness of the hypothesized relationships.

**Fig. 3:**
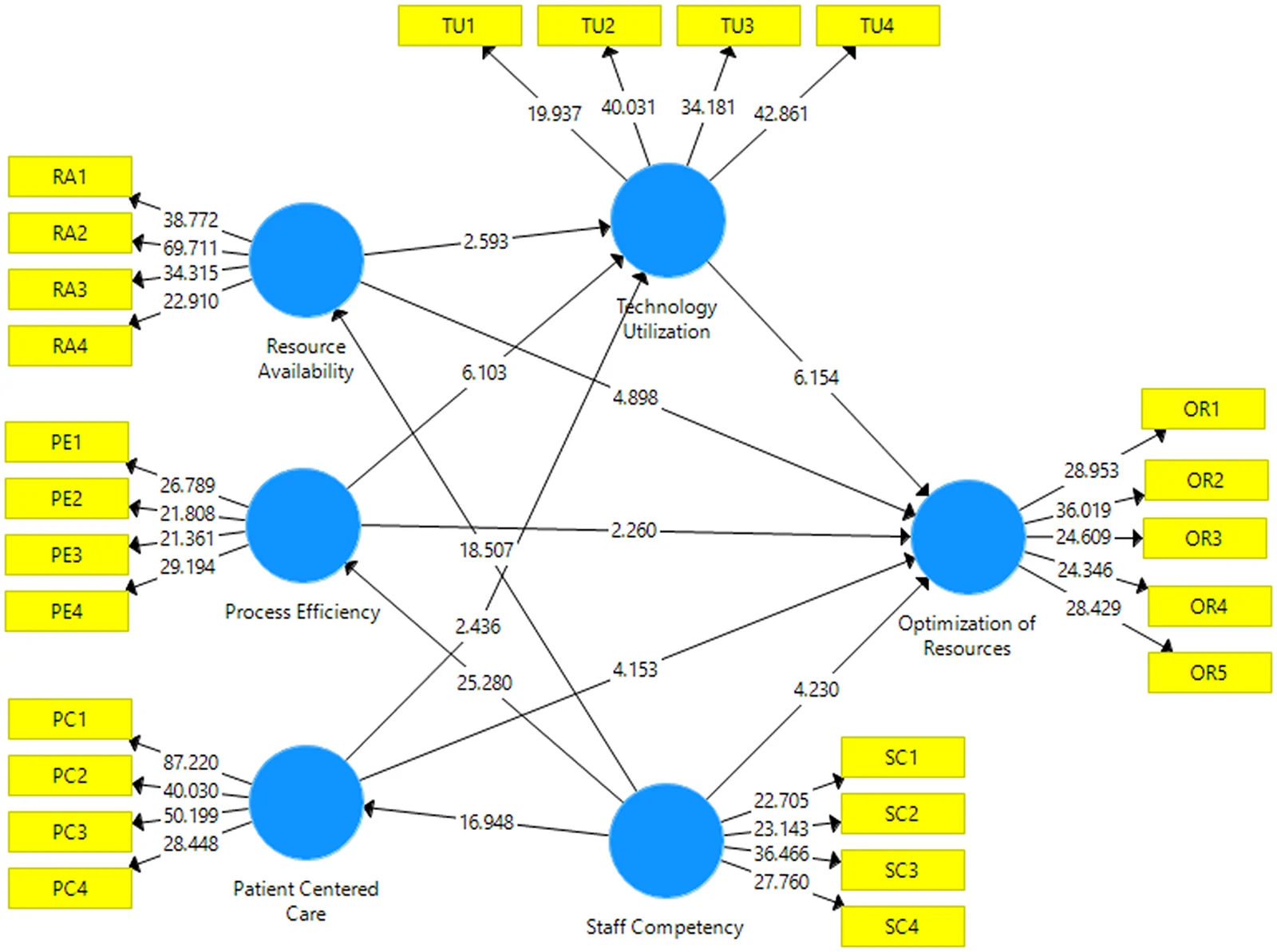
Bootstrapping result (5000)

**Table 6:** Hypothesis test (direct effect)

Hypothesis	Relationship	Beta (β)	St. Dev.	t-value	*p*-values	Supported?
**H1a**	**RA -> OR**	0.218	0.045	4.898	**0.000**	Yes
**H1b**	**RA -> TU**	0.193	0.074	2.593	**0.010**	Yes
**H2a**	**PE -> OR**	0.096	0.043	2.260	**0.024**	Yes
**H2b**	**PE -> TU**	0.382	0.063	6.103	**0.000**	Yes
**H3a**	**PC -> OR**	0.170	0.041	4.153	**0.000**	Yes
**H3b**	**PC -> TU**	0.156	0.064	2.436	**0.015**	Yes
**H4a**	**SC -> OR**	0.249	0.059	4.230	**0.000**	Yes
**H4b**	**SC -> PC**	0.600	0.035	16.948	**0.000**	Yes
**H4c**	**SC -> PE**	0.688	0.027	25.280	**0.000**	Yes
**H4d**	**SC -> RA**	0.630	0.034	18.507	**0.000**	Yes
**H5a**	**TU -> OR**	0.328	0.053	6.154	**0.000**	Yes

**Table 7:** Mediation effect

Hypothesis	Mediation Path	Beta (β)	St. Dev.	t-value	*p*-values	Supported?
**H6a**	**RA -> TU -> OR**	0.063	0.026	2.415	**0.016**	Yes
**H6b**	**PE -> TU -> OR**	0.125	0.031	4.071	**0.000**	Yes
**H6c**	**PC -> TU -> OR**	0.051	0.022	2.305	**0.021**	Yes

**Table 8:** Moderation effect

Hypothesis	Moderation Path	Beta (β)	St. Dev.	t-value	*p*-values	Supported?
**H7a**	**SC -> RA-> OR**	0.137	0.029	4.778	**0.000**	Yes
**H7b**	**SC -> PE -> OR**	0.066	0.030	2.211	**0.027**	Yes
**H7c**	**SC -> PC -> OR**	0.102	0.025	4.116	**0.000**	Yes
**H7d**	**SC-> RA -> TU**	0.122	0.050	2.434	**0.015**	Yes
**H7e**	**SC -> PE -> TU**	0.263	0.048	5.495	**0.000**	Yes
**H7f**	**SC -> PC -> TU**	0.094	0.041	2.299	**0.022**	Yes

### Hypothesis testing, mediation, and moderation results

Table 6 and Fig. 3 present the direct effect hypotheses, all of which were supported. Resource Availability (RA), Process Efficiency (PE), Patient-Centered Care (PC), Staff Competency (SC), and Technology Utilization (TU) positively influenced Optimization of Resources (OR) and/or TU, with β values ranging from 0.096 to 0.688 (*p* < 0.05). SC showed particularly strong effects on PC, PE, and RA, while TU significantly impacted OR. These results confirm that all hypothesized direct relationships are significant and in the expected direction. Mediation analysis (Table 7; Fig. 3) indicated that TU significantly mediated the effects of RA, PE, and PC on OR (RA → TU → OR: β = 0.063, *p* = 0.016; PE → TU → OR: β = 0.125, *p* < 0.001; PC → TU → OR: β = 0.051, *p* = 0.021). This highlights TU’s role as a key mechanism through which these antecedents influence OR. Moderation results (Table 8) demonstrated that Staff Training (ST) strengthened the effects of RA, PE, and PC on both OR and TU (β = 0.066–0.263, all *p* < 0.05), emphasizing that training enhances the impact of these constructs on organizational outcomes. Collectively, these findings provide strong support for the proposed direct, mediating, and moderating relationships, confirming the robustness of the overall model.

## Discussion

Healthcare systems today face increasing pressure to optimize limited resources while simultaneously improving quality, efficiency, and patient outcomes. Achieving this balance requires not only the availability of adequate resources but also the effective design of processes, adoption of patient-centered practices, and strategic use of digital technologies. However, the impact of these factors on resource optimization is not straightforward, as it often depends on how well technology is integrated into daily operations and the competency of staff in leveraging it. Building on the Resource-Based View (RBV), Socio-Technical Systems theory, and the Dynamic Capabilities perspective, this study develops a model that links resource availability, process efficiency, and patient-centered care to the optimization of resource allocation, while considering the mediating role of technology utilization and the moderating effect of staff competency.

The study disclosed that resource availability significantly affecting optimization of resource allocation. Based on this result finding, hospitals with greater and better-targeted resource availability (financial, equipment, beds, supplies) are more able to allocate resources optimally because need-based allocation rules and adequate inputs reduce trade-offs and bottlenecks in service delivery. Alenazi et al., [7] elucidated that medical professionals play a crucial role in optimizing resource allocation within clinical environments, improving patient care despite constraints. Through effective practices, communication, and collaboration, they can enhance efficiency, reduce unnecessary use of resources, and promote patient-centered care, offering practical guidance for administrators and policymakers. Empirical and review studies on need-based indicators and regional allocation show that the distribution and adequacy of resources are foundational to rational allocation decisions and service efficiency.

The study results disclosed that process efficiency has a significant effect on optimization of resource allocation. In line with this finding, a study by Abu Saq et al. [1] confirmed that effective leadership plays a crucial role in optimizing resource allocation within hospitals. Approaches such as transformational and servant leadership encourage collaboration, reduce inefficiencies, and align resources with patient needs. By fostering teamwork and supporting patient-centered care models, leaders help minimize waste and improve both staff performance and patient outcomes. This suggests that leadership commitment is a key driver in ensuring that scarce resources are used effectively to deliver quality care. A study made by Al Amin et al. [4] disclosed also that improvements in internal processes such as workflow standardization, patient flow, and scheduling increase throughput and reduce waste, directly improving how scarce resources are used. Studies of hospital efficiency consistently find that process redesign and operational improvements raise capacity and reduce unnecessary utilization, enabling better allocation of staff, equipment and beds [3]. These findings highlight that process efficiency is central to resource optimization. Small improvements in workflows and scheduling can free up staff time and reduce waste, while leadership support ensures such changes are sustained. This shows that efficiency is not only a technical issue but also a cultural one, requiring both system redesign and committed leadership to make the best use of limited resources.

The study confirms that patient-centered care significantly enhances resource allocation. Supporting this, Binsar et al. [21] show that approaches such as care planning, self-management support, and shared decision-making reduce unnecessary admissions, shorten length of stay, and target services to patient needs, thereby freeing up resources. Similarly, Alshehri et al. [11] highlight that patient-centered care minimizes waste, avoids duplication, and directs resources where most needed, with team collaboration and digital tools further enhancing efficiency despite persistent communication gaps. These findings align with the perspective that healthcare is most effective when it begins with the patient: by focusing on actual patient needs, hospitals can reduce unnecessary treatments and delays, saving time, costs, and effort. Thus, embedding patient-centered care not only improves outcomes but also optimizes the use of limited resources, benefiting both patients and healthcare providers [63].

The findings highlight that technology is not merely a supporting tool but a critical mediator in translating resource availability, process efficiency, and patient-centered practices into optimized resource allocation. While inputs and processes are necessary, their impact depends heavily on the effective adoption of digital solutions. Bachina and Kanagala [16], show that health information technologies improve coordination, reduce duplication, and enable data-driven resource assignment, while Dordunoo [30] finds that digital capability can enhance patient-centered care even in lower–mid-level hospitals. Leadership support and skilled ICT staff emerge as key enablers, whereas limited ICT budgets remain a major barrier, preventing hospitals from fully realizing digital health benefits [24, 57]. Overall, technology serves as the mechanism through which resources and process improvements translate into measurable gains in allocation efficiency and care quality.

The findings underscore that staff competency is a critical moderator in leveraging technology for resource optimization and enhancing patient-centered care. While technologies such as AI offer significant potential to improve operational efficiency, cost management, and staffing responsiveness [31, 50, 56], their effectiveness depends heavily on the skills and digital literacy of healthcare personnel. As highlighted by Zeb et al. [66], staff must possess both clinical and digital competencies to interpret analytics, utilize decision-support systems accurately, and adapt workflows; without these skills, technology can be underutilized or misapplied, limiting its impact. This suggests that investments in technology alone are insufficient organizations must concurrently develop staff competencies to fully realize gains in resource allocation, process efficiency, and patient-centered outcomes. Falade et al. [33] confirmed that targeted strategies such as ICU handover simulations, standardized protocols, interprofessional communication, and virtual education significantly enhance patient safety and care quality. Innovations like machine learning and zero-harm programs further support proactive, evidence-based, and patient-centered care [60]. Overall, interdisciplinary collaboration, continuous training, and integrated quality improvement are essential for fostering a resilient safety culture and optimizing healthcare outcomes. In essence, the transformative potential of technology in healthcare is contingent upon the human capacity to harness it effectively.

## Conclusion

This study concludes that optimizing resource allocation in healthcare organizations requires an integrated approach that combines adequate resource availability, efficient operational processes, patient-centered care, and effective technology utilization. The findings demonstrate that resource availability, process efficiency, and patient-centered care are significant determinants of resource optimization, indicating that hospitals can achieve better allocation outcomes by ensuring sufficient inputs, improving workflows, and aligning healthcare services with patient needs. However, these factors alone are insufficient to guarantee optimal resource utilization; rather, their effectiveness depends largely on the extent to which digital technologies are effectively adopted and embedded within healthcare operations.

The study further confirms the mediating role of technology utilization, highlighting that digital health solutions serve as an essential mechanism through which organizational resources, efficient processes, and patient-focused practices are transformed into improved allocation decisions and healthcare performance. Moreover, staff competency strengthens this relationship by enabling healthcare professionals to effectively utilize technological systems, interpret data-driven insights, and integrate digital tools into clinical and administrative practices. Therefore, technology investments must be accompanied by continuous capacity development, digital literacy enhancement, and supportive organizational environments to maximize their benefits.

From a theoretical perspective, this study extends the Resource-Based View, Socio-Technical Systems theory, and Dynamic Capabilities perspective by demonstrating that healthcare resource optimization is not solely dependent on tangible resources but also on the interaction between organizational capabilities, technological infrastructure, and human competencies. Practically, the findings suggest that hospital administrators and policymakers should adopt holistic strategies that combine resource planning, process improvement, patient-centered approaches, digital transformation initiatives, and workforce development. Such an integrated framework can help healthcare organizations overcome resource constraints, improve operational efficiency, and deliver sustainable, high-quality, and patient-focused healthcare services.

## Theoretical contributions

This study makes several contributions to theory by extending and integrating perspectives from the Resource-Based View (RBV), Socio-Technical Systems (STS), and Dynamic Capabilities Theory within the context of healthcare resource management. First, it advances RBV by showing that intangible resources, such as staff competency, and strategic capabilities, such as technology utilization, are not just supportive assets but critical drivers in optimizing scarce healthcare resources. This highlights that hospitals, particularly in resource-constrained environments, can achieve efficiency not only through physical assets but also by leveraging human and technological capital.

Second, the study enriches STS theory by illustrating how the interplay between people and technology influences resource allocation. By positioning technology utilization as a mediator, the findings demonstrate that technical systems alone are insufficient; their effectiveness depends on how well healthcare staff integrate and apply them within daily processes. This reinforces the idea that organizational outcomes emerge from the alignment of social and technical subsystems.

Third, the study contributes to the dynamic capabilities literature by emphasizing how hospitals in developing contexts reconfigure resources to adapt to systemic challenges. The moderating effect of staff competency shows that dynamic learning and adaptability are essential for sustaining performance when financial and infrastructural resources are limited. Thus, the framework underscores how hospitals build resilience by combining competencies and technology to continually optimize resource use. Generally, the study adds to healthcare management theory by positioning resource optimization as not only an operational challenge but also a theoretical construct shaped by the interaction of resources, capabilities, and contextual constraints. This integrative perspective advances understanding of how hospitals can achieve efficiency while maintaining a patient-centered orientation.

## Practical contributions

This study provides several practical insights that can guide healthcare managers, policymakers, and practitioners working in resource-constrained environments such as Ethiopia. First, the findings emphasize the importance of strengthening staff competency. Hospitals should invest in continuous professional development, interdisciplinary training, and leadership support to ensure that healthcare workers can effectively translate limited resources into improved patient outcomes.

Second, the study highlights the mediating role of technology utilization. This suggests that investment in digital systems such as electronic medical records, patient monitoring tools, and data-driven planning platforms can significantly improve the alignment between patient needs and available resources. However, such investments must be matched with adequate ICT training and supportive infrastructure to ensure successful implementation.

Third, the study underscores the practical value of patient-centered care and process efficiency in resource optimization. Hospitals that engage patients in care planning, reduce workflow bottlenecks, and eliminate unnecessary duplication are better positioned to minimize waste and maximize service delivery.

Finally, the results offer practical direction for policy and hospital governance. Policymakers can use these insights to design policies that integrate human resource development, technology adoption, and patient-centered models into national healthcare strategies. For hospital leaders, the framework provides actionable guidance on how to balance scarce resources while maintaining high standards of care.

## Limitations and directions for future studies

Like all empirical studies, this research is not without limitations. First, the study focused on hospitals in Ethiopia, a resource-constrained setting. While this provides valuable insights, it also limits the generalizability of the findings to high-income countries with different healthcare structures and resource availability. Future studies could undertake comparative research across diverse health systems to explore whether the mediating and moderating effects identified here hold in varied contexts.

Second, the study relied primarily on cross-sectional survey data, which restricts the ability to establish causal relationships between the constructs. Longitudinal or experimental designs would provide stronger evidence of how staff competency and technology utilization interact with organizational processes over time.

Third, the study measured constructs such as patient-centered care and process efficiency through self-reported perceptions, which may be subject to respondent bias. Incorporating objective performance metrics such as hospital readmission rates, patient wait times, or cost data would help validate the findings and reduce measurement bias.

Finally, while technology utilization was examined as a mediator and staff competency as a moderator, other potentially influential variables such as leadership style, organizational culture, or regulatory frameworks were not included. Future research could expand the model to test additional factors that may influence how hospitals optimize resource allocation in complex healthcare environments. In summary, addressing these limitations in future research will deepen understanding of how hospitals can leverage human and technological capabilities to achieve sustainable resource optimization and improved patient outcomes.

## Data Availability

The data supporting the findings of this study were collected and analyzed by the authors and are not publicly available. The datasets and related research materials contain no confidential or proprietary information and can be obtained from the corresponding author upon reasonable request.
